# Biocontrol Effect and Possible Mechanism of Food-Borne Sulfide 3-Methylthio-1-Propanol Against *Botrytis cinerea* in Postharvest Tomato

**DOI:** 10.3389/fpls.2021.763755

**Published:** 2021-12-14

**Authors:** Shun Feng, Wang Lu, Yongfei Jian, Yu Chen, Run Meng, Jie Deng, Qing Liu, Tingting Yu, Liang Jin, Xingyong Yang, Zhengguo Li, Wei Jian

**Affiliations:** ^1^School of Life Sciences, Chongqing Normal University, Chongqing, China; ^2^Key Laboratory of Plant Hormones and Development Regulation of Chongqing, School of Life Sciences, Chongqing University, Chongqing, China; ^3^College of Bioengineering, Chongqing University, Chongqing, China

**Keywords:** *Botrytis cinerea*, 3-methylthio-1-propanol, antifungal activity, fumigation, sulfide

## Abstract

*Botrytis cinerea* is one of the most destructive fungal pathogens causing tremendous losses in fresh fruit or vegetables. 3-Methylthio-1-propanol (3-MP) is a naturally occurring food-borne sulfide, which is mainly used to increase the flavor in food. However, the potential application of 3-MP in the postharvest phase to manage fruit fungal diseases has not been explored. In this study, the antifungal activity of 3-MP against *B. cinerea* was evaluated, and the possible mechanism involved was explored. *In vitro* 3-MP treatment could effectively inhibit the mycelial growth, spore germination, and germ tube elongation of *B. cinerea.* 3-MP also impaired the spore viability and membrane integrity of *B. cinerea* as well as increased the leakage of nucleic acids, proteins, and malondialdehyde (MDA) in *B. cinerea*. *In vivo* 3-MP fumigation treatment inhibited the infection of *B. cinerea* on tomato fruits. Also, the fruits with 3-MP fumigation treatment exhibited higher antioxidant enzyme activity, lower MDA content, and a significant delay of induction of the expression of most of the stress-related genes when compared to the control group. Moreover, a cytotoxicity evaluation revealed that 3-MP had no toxicity to normal cells in a certain concentration range. Collectively, our research results will provide evidence for the development of food-borne sulfide 3-MP as a fungicide in food and agriculture and will provide an important reference for the formulation of *B. cinerea* biocontrol strategies.

## Highlights

- 3-MP inhibited the infection and growth of *Botrytis cinerea in vitro* and *in vivo.*
- 3-MP fumigation treatment destroyed mycelial structure of *Botrytis cinerea.*
- 3-MP impaired the cell viability and membrane integrity of *Botrytis cinerea.*
- 3-MP affected the defense systems of tomato fruit.
- 3-MP had no toxicity to normal cells.

## Introduction

*Botrytis cinerea* is one of the most serious and widespread postharvest pathogens of much fresh fruit, vegetables, and ornamentals (Chaouachi et al., 2021). *Botrytis cinerea* is recognized as a “high-risk” pathogen for its characteristics of the short life cycle, high reproduction, and genetic variation, which are not beneficial for maintaining the resistance of fungicides (Haidar et al., 2016; Saito et al., 2019). At present, several control strategies have been applied to control *B. cinerea*. Among them, chemical fungicides were considered to be the most widely and economical measure. However, many hazards or risks have been caused by the excessive application of chemical fungicides, mainly such as pollution of the environment, food safety threats, and the production of fungal-resistant strains (Tian et al., 2016; Morita et al., 2019; Liu X. et al., 2020). Hence, there is an urgent need for safe bioderived antimicrobial agents to control *B. cinerea* and reduce the loss of postharvest fruit.

Recently, a large number of bioderived antimicrobial agents that were generally regarded as safe were demonstrated to be effective in controlling postharvest pathogens, such as carvacrol (Pei et al., 2020), methyl thujate (Ma et al., 2020), and others. Among them, a kind of sulfur-containing spice compound was noticeable, which was mainly obtained from plants or microorganisms (Schöller et al., 2002; Hongman et al., 2018; Xie et al., 2020). The study in pear showed that the infection and development of black spots caused by *Alternaria alternata* were effectively inhibited by benzyl isothiocyanate (BITC) fumigation (Wang, 2020). The *in vivo* study showed that both dimethyl disulfide (DMDS) and dimethyl trisulfide (DMTS) exhibit excellent antifungal activity, which can completely inhibit the infection and growth of blue mold on citrus fruits (Wang et al., 2021). In addition, the growth of *Colletotrichum gloeosporioides*, *Aspergillus ochraceus*, *Aspergillus flavus*, and *Sclerotinia minor* could be effectively inhibited by DMTS, and further study indicated that the mechanism involved in this process was mainly due to the suppression of mycelial growth and spore germination with the destruction of the cell membrane (Zuo et al., 2017; Tyagi et al., 2020). Consistently, a recent study revealed the wide use of sulfur-containing spice compounds in controlling fungal diseases in various fruit or vegetables: its excellent role is attributed to the antioxidant and antimicrobial activity (Shang et al., 2019). Therefore, given the uniqueness and effectiveness of food-borne volatile sulfur compounds, more food-borne sulfides should be discovered as microbial inhibitors.

The existed studies indicated that 3-methylthio-1-propanol (3-MP) was first reported in the volatile flavor compounds in wine (0.86–4.91 mg L^–1^) (Schreier et al., 1974; Fedrizzi et al., 2007; González-Álvarez et al., 2012). 3-MP was found widely in some fruit, beer, and malt whisky as a natural sulfur-containing spice compound (Etschmann et al., 2008; Moreira et al., 2010). A previous report showed that 3-MP can significantly contribute to the flavor quality of wine, bread, and other foods (Moreira et al., 2011) and can enhance the saltiness of weak salt content and the umami taste of monosodium glutamate solution (Zhou et al., 2021). To our knowledge, whether 3-MP has an antimicrobial activity is still unknown so far. Given this, the objective of this study was to investigate the inhibitory effect of 3-MP against *B. cinerea* by conducting *in vitro* and *in vivo* assays, and the possible mechanisms involved in this process were also explored through microscopic observation, physiological detection, and gene expression analysis. The results obtained would lay a foundation for the development of 3-MP as a new fungicide and also provide an important reference for developing an eco-friendly strategy to control *B. cinerea*.

## Materials and Methods

### Materials and Chemicals

The fungal pathogen *B. cinerea* B05.10 used in this study was obtained from the School of Life Sciences, Chongqing University, China. Cherry tomato (*Solanum lycopersicum*) fruits that were free from wounds with comparable size and stage of maturity were harvested from Shiyi Bai experimental orchard for infection experiments.

For the chemicals, 3-MP can be easily dissolved into the water and purchased from Sigma (St. Louis, MO, United States). The fluorescein diacetate (FDA, Yeasen Biotechnology Co., Ltd., Shanghai, China) and propidium iodide (PI, Yeasen Biotechnology Co., Ltd., Shanghai, China) were purchased from Sangon Biotechnology Co., Ltd. (Shanghai, China).

### Evaluation *in vitro* and *in vivo* of the Antifungal Activity of 3-Methylthio-1-Propanol Fumigation Treatment

For *in vitro* assay, *B. cinerea* mycelial plugs were placed on the side of the two-part plates containing potato dextrose agar (PDA) medium, and 0, 10, 25, 50, 100, and 200 μl of 3-MP was added to the other side of the two-part plates. After inoculation, the Petri dishes were quickly sealed and placed in 25°C constant temperature incubators for 5 days, and then, the colony diameters were measured and recorded. According to the modified method of Elsherbiny et al. (2020), the effects of 3-MP on mycelium morphology were analyzed. The mycelium exposed to 3-MP treatment was fixed with 2.5% glutaraldehyde for 2 h and washed three times for 10 min in 0.1 M phosphate buffer (pH 7.0). After fixation, the samples were dehydrated in graded ethanol series (30, 50, 70, 80, 90, and 100%), for 15 min in each process. The samples were then washed in 3:1, 1:1, and 1:3 mixtures of ethanol and tert-butyl alcohol and finally dipped into pure tert-butyl alcohol, for 10 min in each process. The samples were dried by sublimation in a freeze dryer (ES-2030; HITACHI, Japan) and coated with gold by a sputter coating (E1010, HITACHI, Japan) for 30 s. The samples of the same treatment were sampled three times independently and observed after preparation. A scanning electron microscope (TM4000Plus II, HITACHI, Japan) was used to observe the morphological characteristics of *B. cinerea* mycelium.

**FIGURE 1:**
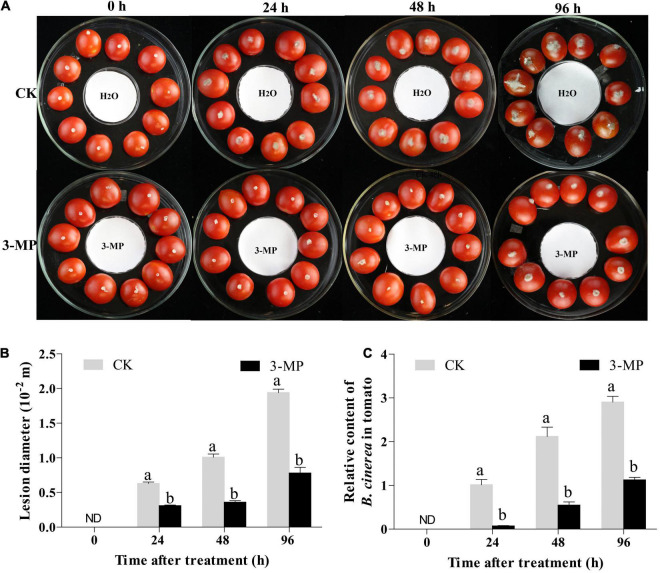
3-Methylthio-1-propanol (3-MP) decreased the symptoms of gray mold in tomato fruit. **(A)** Disease symptoms on tomato treated with sterile water or 3-MP for 0, 24, 48, and 96 h. **(B)** Effect of 3-MP on lesion diameter of gray mold. **(C)** Accumulation of *Botrytis cinerea* in fruit after inoculation for 0, 24, 48, and 96 h. ND means not detection. *B. cinerea* content was calculated by the comparison of the ratio of *B. cinerea* DNA to tomato DNA. The experiment was performed in triplicates, and values represent the means ± SD of 10 individual fruit. The different lowercase letter means statistically significant (Student’s *t*-test).

For *in vivo* assay, tomato fruits that were uniform in size and disease-free were washed and dried, and 10 fruits were placed in each large glass Petri dish (15 cm in diameter and 5.5 cm in height). After the tomato (equatorial position) was scratched by a sterile toothpick, a gray-mold mycelial plug (3.0 mm) was inoculated on the tomato wound. Then, a small Petri dish (i.e., 6.0 cm in diameter, with a piece of filter paper) was placed in the center of the large glass Petri dish (Figure 1A). The Petri dishes were immediately sealed after adding 50 μl 3-MP or sterile water to the filter paper and placed in a 25°C constant temperature incubator for cultivation. Each treatment consisted of 12 large Petri dishes. Tomato lesions were monitored at 0, 24, 48, and 96 h following treatment. For sampling collection, the fruit tissue with a diameter of 1.5 cm around the inoculation point was collected after removing the mycelial plugs on the surface of the tomato, and the tissues of every 10 fruits were gathered and mixed, put into liquid nitrogen immediately, and finally stored at –80°C for further use. This experiment was repeated three times independently from fumigation treatment to sampling.

### Extraction of Total RNA, DNA, and Quantitative Real-Time PCR

Total RNA was extracted from tomato fruits with different treatments using an RNeasy Plant Mini Kit (Tiangen, China) according to the protocol of the manufacturer.

The DNA of tomato fruits with different treatments was extracted using a Plant Genomic DNA Kit (CWBio, China) to quantify the amount of gray mold.

To measure the expression level of fungal pathogenicity genes, *B. cinerea* was cultured as the method described earlier (Wang et al., 2020). In brief, mycelium was collected after culturing in potato dextrose broth (PDB) medium containing 0, 25, and 100 μl L^–1^ 3-MP for 12 h.

For the quantitative real-time PCR (qRT-PCR) assay, NanoDrop Lite (Thermo Scientific, California, United States) was used to detect the centration and quality of the total RNA or DNA. The first-strand cDNA was synthesized by using PrimeScript™ RT Reagent Kit with gDNA Eraser (Perfect Real Time) (TAKARA, Japan). The qRT-PCR mixture was performed using an SYBR^®^ Premix Ex Taq™ (Tli RNaseH Plus) (TAKARA, Japan), and the reaction was conducted with a Bio-Rad CFX system (Bio-Rad, California, United States). Each sample was performed in triplicate, and the relative fold differences were calculated using a comparative Ct method. *SlUBI* and *BcActin* were used as the internal reference in tomato and *B. cinerea* background, respectively (Hua et al., 2019; Jian et al., 2019). All the primers used in the whole experiment were listed in Supplementary Table 1.

### Measurement of Antioxidant Enzymes Activity

The tissue sample (3.0 g) was weighed and then placed together with less silica sand (0.3 g) in a prefrozen mortar, and 10 ml precooled phosphate-buffered saline (PBS; 50 mM, pH 7.8) with 1% polyvinylpyrrolidone was added to grind gently (Zhao et al., 2020). The ground tissue sample that was obtained above was fully mixed with 9 ml of PBS and then transferred to a centrifuge tube for centrifugation at 12,000 × *g* for 8 min at 4°C. Then, the supernatant was collected to evaluate the activity of antioxidant enzyme Superoxide Dismutase (SOD), Peroxidase (POD), and Catalase (CAT) according to the operating instruction provided by the Nanjing Construction Bioengineering Research^1^ kit (Liu Y. et al., 2020). The experiment was repeated two times, and each treatment was conducted in triplicate. All the tomato tissue weights that were mentioned in the whole manuscript represent the fresh weight. Enzyme activity is defined as U kg^–1^ of fresh weight.

### Cytoplasmic Leakage Assay

The spore suspension of *B. cinerea* (10^7^ spores per ml) was inoculated in a PDB medium placed in a shaker incubator with the condition of 180 rpm, 25 ± 1°C (Wang et al., 2020). After 3 days, 2.0 g mycelium was collected with filter paper and resuspended in sterile distilled water containing 3-MP (0, 25, 100 μl L^–1^). After incubation at 25 ± 1°C for different times (i.e., 0, 3, 6, 9, and 12 h), the supernatant was collected for the detection of cytoplasmic leakage. For the measurement of leakage of nucleic acids, the optical density at 260 nm (OD_260_) of the supernatant was measured. The leakage of proteins was measured with a Bradford Protein Assay Kit (Nanjing Construction Bioengineering Research). The malondialdehyde (MDA) content was tested by the method of thiobarbituric acid (TBA) (Pereira et al., 2003). The experiment was repeated two times, and each treatment was conducted in triplicate.

### Fluorescence Microscopy

Both FDA and PI were used to examine the spore viability and membrane integrity by counting stained spores (Wang et al., 2020). After being in a PDB medium with 3-MP (0, 25, 100 μl L^–1^) for 3 h at 25°C, the spores were treated with 50 mg L^–1^ FDA and 20 mg L^–1^ PI for 10 min at 25°C, respectively, and then observed by using a fluorescence microscope (Leica, Germany). Spores were counted using a hemocytometer. Five fields of view were randomly observed per treatment, and the experiment was repeated two times.

### Effects of 3-Methylthio-1-Propanol on Cell Viability and the Measurement of Half-Maximal Inhibitory Concentration

To evaluate the security and application potential of 3-MP, a cytotoxicity assay was conducted. 3-MP was diluted to 0, 2, 4, 8, 16, 32, 64, 128, 256, and 512 mg L^–1^, respectively, with cell culture medium (Gibco, California, United States). Mouse fibroblast cells (L929), human non-small cell lung cancer cells (A549), and mouse breast cancer cells (4T1) were chosen in this experimental section. Notably, 100 μl cell suspension (about 10,000 cells) was added to 96-well plates, and three repeated experiments were set at each concentration. Cells were cultured in the incubator for 24 h (37°C and 5% CO_2_), and then, 10 μl each of different concentrations of 3-MP was added. The effects of 3-MP on cell viability were measured according to the Cell Counting Kit-8 (CCK8) method (Bioground, China). Later, cells were cultured in the incubator for 12 h. Finally, the viability of three different cells was analyzed to acquire the IC50 value of 3-MP.

### Statistical Analysis

The statistical tests were performed using SPSS software (version 17.0) (IBM, New York, United States) with a one-way ANOVA. Duncan’s multiple range test and Student’s *t*-tests were used to analyze the data, and a difference was considered to be statistically significant when *P* < 0.05.

## Results

### 3-Methylthio-1-Propanol Inhibited the Growth of *Botrytis cinerea in vitro*

**FIGURE 2:**
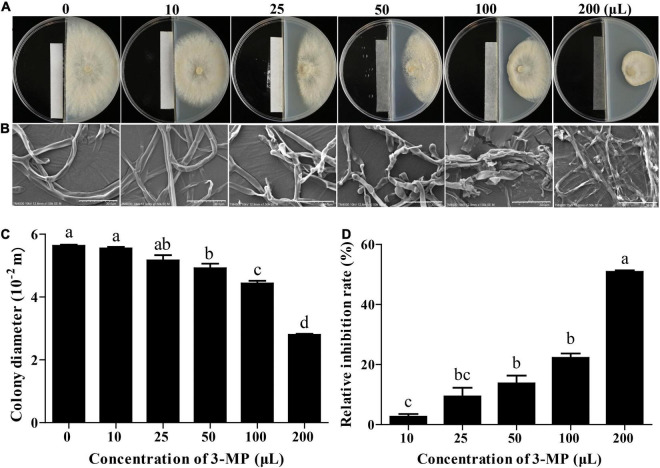
The antifungal ability of 3-methylthio-1-propanol (3-MP) against *B. cinerea* on PDA medium. Phenotypic **(A)** and scanning electron microscopic **(B)** characterization of colony expansion of *B. cinerea* treated with or without 3-MP fumigation treatment. Bar = 30 μm. Detection of colony diameter **(C)** and relative inhibition rate **(D)** in control and 3-MP fumigation treatment group. Values are the means ± SD of three biological replicates analyzed by Duncan’s multiple range test. The different lowercase letter means statistically significant.

The mycelial growth of *B. cinerea* was gradually increased in the control plates throughout the incubation period. However, the mycelial growth in the 3-MP fumigation treatment was significantly inhibited and exhibited a dose-dependent fashion (Figure 2A). The 3-MP fumigation treatment also destroyed the ultrastructure of *B. cinerea* mycelium. The degree of mycelial damage is positively correlated with the concentration of 3-MP (Figure 2B). Especially at 5 days following treatment, in comparison with the control group, the mycelial growth was significantly inhibited to 13.7, 22.2, and 50.8% by 50, 100, and 200 μl 3-MP, respectively (Figures 2C,D).

**FIGURE 3:**
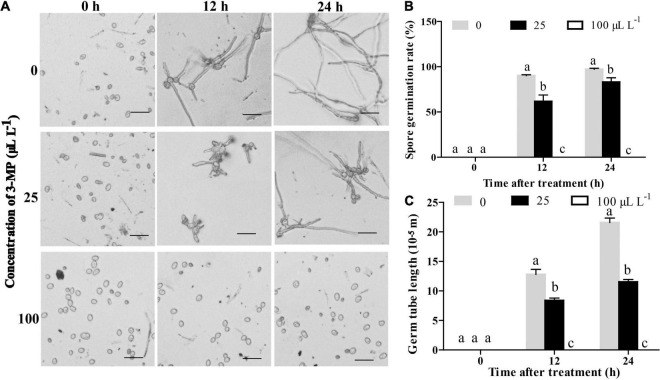
Analysis of the antifungal role of 3-MP on *B. cinerea* germination and growth. **(A)** Phenotypic characterization of spore germination of *B. cinerea* treated with sterile water or different concentration of 3-MP for 0, 12, and 24 h. Bar = 40 μm. **(B)** The spore germination rate and **(C)** the germ tube elongation of *B. cinerea* treated with sterile water or different concentration of 3-MP for 0, 12, and 24 h. Approximately 100 spores were randomly observed in each treatment. The experiment was performed in triplicates, and the values represent means ± SD. The different lowercase letter means statistically significant.

To further analyze the physiological changes, the influence of 3-MP on spore germination and germ tube elongation of *B. cinerea* was observed. The results indicated that the spore germination and germ tube elongation of *B. cinerea* was suppressed by treatment of 3-MP (Figure 3). At 12 h following treatment, more than 90.2% of the spores germinated on the control media, while the germination rate was decreased to 61.6% on the media treated by 25 μl L^–1^ 3-MP. Also, it is worth specifically mentioning that the spore germination and germ tube elongation of *B. cinerea* was completely suppressed by 100 μl L^–1^ 3-MP.

### 3-Methylthio-1-Propanol Fumigation Treatment Decreased the Disease Phenotype of Gray Mold in Tomato Fruit

To evaluate whether 3-MP fumigation treatment has an antifungal activity on *B. cinerea in vivo*, the tomato fruit was chosen in this study. As shown in Figure 1A, the lesions in tomato fruits developed after inoculation for 24 h and the lesions in the control fruits were significantly larger than that 50 μl 3-MP treatment. Especially at 96 h following incubation, the diameter of the lesion in 3-MP-treated fruit was 7.8 mm, while the diameter of the lesion in the control fruit was 1.94 mm, which was 2.49-fold higher than the former (Figure 1B). Consistent with this, the quantitative results of qPCR showed that the accumulation of *B. cinerea* in control fruits was significantly higher than that in 3-MP-treated fruits at all the monitoring times (Figure 1C). In addition, we also detected the content of 3-MP in the fumigated tomato fruit. The results showed that the content of 3-MP in the fumigated tomato fruit was 1.6–2.24 mg L^–1^ (Supplementary Figure 1). These results indicated that the fumigation treatment of 3-MP can greatly inhibit the development of rot disease caused by *B. cinerea*.

### 3-Methylthio-1-Propanol Impaired Spore Viability and Plasma Membrane Integrity of *Botrytis cinerea*

**FIGURE 4:**
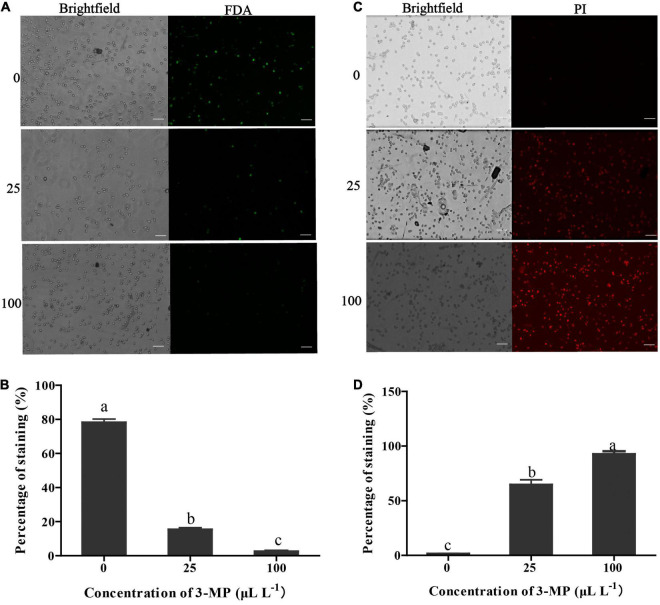
Evaluation of the spore viability and membrane integrity of *B. cinerea*. **(A)** Micro-observation of the spores stained with FDA. Bar = 50 μm. **(B)** The percentage of stained spores with fluorescein diacetate (FDA). **(C)** Micro-observation of the spores stained with propidium iodide (PI). Bar = 50 μm. **(D)** The percentage of stained spores with PI. Values are the means ± SD of three biological replicates. The different lowercase letter means statistically significant (Duncan’s multiple range test).

The percentage of *B. cinerea* spores stained by FDA in the control was 78.16% (Figures 4A,B), suggesting that most of the fungal spores were survival. However, the percentage of staining spores in 3-MP treatment was 15.27 and 2.47%, suggesting that most of the fungal spores were dead. It has been suggested that PI can penetrate the plasma membrane of dead spores that have lost membrane integrity and can be stained with red fluorescence. As shown in Figures 4C,D, in comparison with the control, more spores were stained by PI after treatment of 25 or 100 μl L^–1^ 3-MP (64.57 and 92.56%, respectively), implying that most spores lost membrane integrity. These results indicated that both the spore survival and membrane integrity of *B. cinerea* were greatly inhibited by treatment of 3-MP.

### Determination of Cytoplasmic Leakages and Membrane Lipid Peroxidation

**FIGURE 5:**
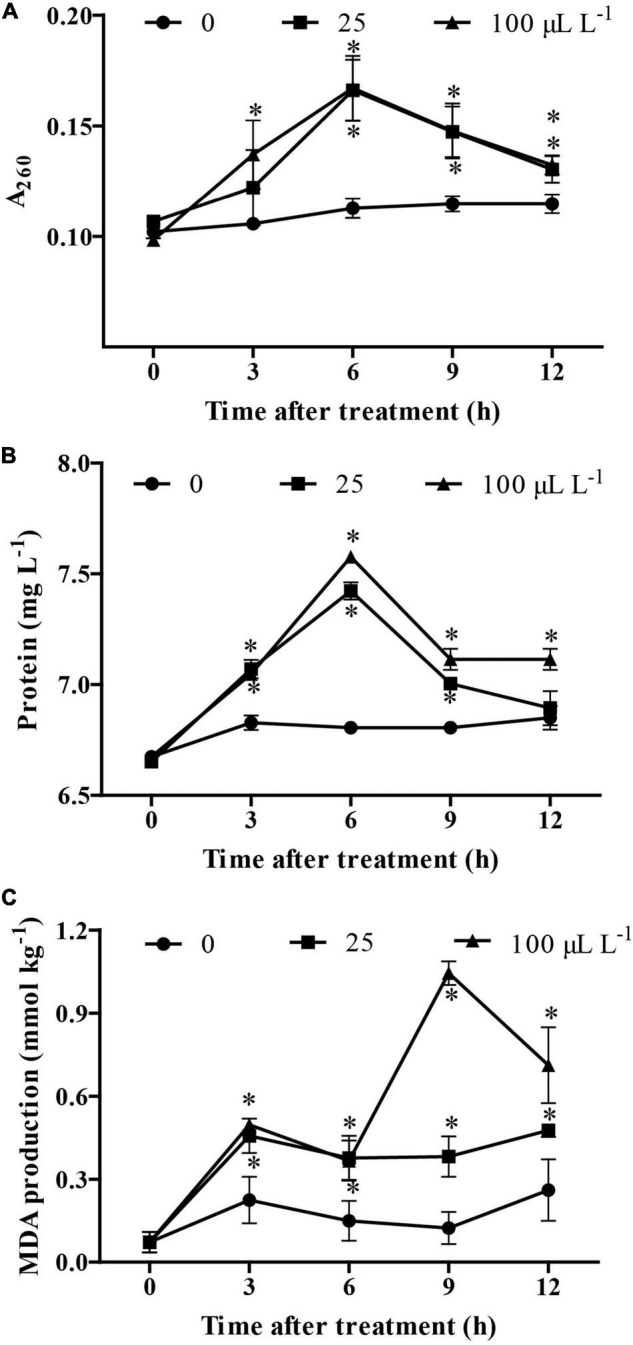
Measurement of cytoplasmic leakages and lipid peroxidation of *B. cinerea*. **(A)** Nucleic acids, **(B)** soluble proteins, and **(C)** malondialdehyde (MDA) content were determined. Values are the means ± SD of three biological replicates. *P < 0.05.

The results showed that, at 3 h following treatment, an increase in the content of nucleic acids and proteins was observed in *B. cinerea* treated by 3-MP when compared to the control group. Noteworthy, at 6 h following treatment, the content of nucleic acids and proteins in the culture medium reached the highest value, suggesting that most of the mycelium had been disrupted by treatment of 3-MP (Figures 5A,B). Consistently, although the MDA content in the control group was relatively stable at all the monitoring times, the MDA content in *B. cinerea* treated by 3-MP was higher than that in the control group at all the corresponding monitoring times (Figure 5C). These results indicated that 3-MP can impair the membrane integrity of *B. cinerea* mycelium, which resulted in cytoplasmic leakages and membrane lipid peroxidation of *B. cinerea*.

### 3-Methylthio-1-Propanol Downregulated the Expression Level of Genes Related to Pathogenicity

**FIGURE 6:**
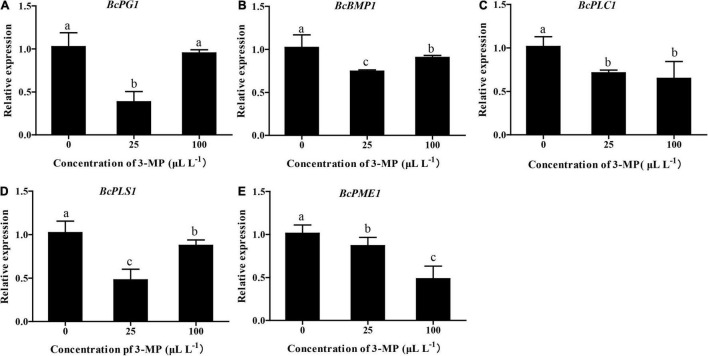
Evaluation of the expression level of genes related to pathogenesis. **(A)** *BcPG1*, **(B)** *BcBMP1*, **(C)** *BcPLC1*, **(D)** *BcPLS1*, and **(E)** *BcPME1* genes involved in the virulence and infection process of *B. cinerea* were selected. The experiment was performed in triplicates, and values represent means ± SD. The different lowercase letter means statistically significant (Duncan’s multiple range test).

To evaluate whether the virulence or ability of infection of *B. cinerea* was affected by 3-MP, the expression level of *BcPG1*, *BcBMP1*, *BcPLC1*, *BcPLS1*, and *BcPME1*, which are key genes in determining the infection and growth of *B. cinerea*, was analyzed (Gourgues et al., 2004; Schumacher et al., 2008; Cong et al., 2019). As shown in Figure 6, the qRT-PCR results indicated that the expression level of all these genes was differentially downregulated in the 3-MP-treated group. The expression level of *BcPG1*, *BcBMP1*, and *BcPLS1* was markedly downregulated by treatment of 25 μl L^–1^ 3-MP but exhibited a slight increase when the concentration of 3-MP reached 100 μl L^–1^. Interestingly, the expression level of *BcPLC1* was greatly downregulated when treated by 25 μl L^–1^ 3-MP but not further changed when the concentration of 3-MP reached 100 μl L^–1^. In addition, 3-MP greatly downregulated the expression level of *BcPME1* and exhibited a negative correlation with the concentration of 3-MP.

### The Antioxidant Enzymes Activity Was Promoted by 3-Methylthio-1-Propanol in Tomato Fruit

**FIGURE 7:**
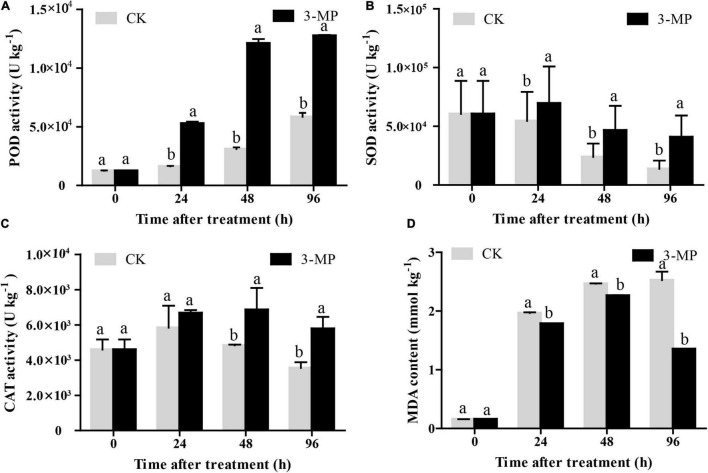
Analysis of the activity of **(A)** POD, **(B)** SOD, **(C)** CAT, and **(D)** MDA content in tomato fruit. Values are the means ± SD of three biological replicates. The different lowercase letter means statistically significant (Student’s *t*-test).

As shown in Figure 7A, the POD activity in control and 3-MP-treated fruits showed a similar trend, both of their activity increased as time went on, and the activity of POD in fruits treated by 3-MP was significantly higher than that in the control fruits at all the monitoring times. Consistently, the changing pattern of the activity of SOD and CAT was similar, both of their activity exhibited a slight increase at first and then a decrease. Also, both of their activities increase to a maximum peak at 24 and 48 h subjected to treatment of 3-MP, respectively. Notably, the activity of SOD and CAT in 3-MP-treated fruits was always higher than that in control fruits during the whole time (Figures 7B,C). Furthermore, the MDA content in 3-MP-treated fruits increased all the time, and it was significantly lower when compared to that in the control fruits at all the monitoring times (Figure 7D). These results indicated that the antioxidant systems in tomatoes were activated by treatment of 3-MP.

### Measurement of the Expression Level of Defense-Related Genes in Tomato Fruit

**FIGURE 8:**
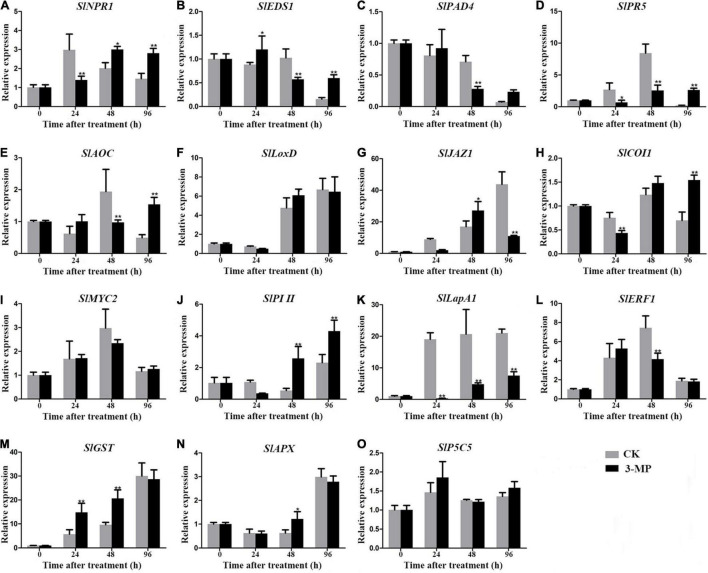
Detection of the expression level of stress-related genes in tomato fruit treated with or without 3-MP. Four salicylic acid signaling-related (*SlNPR1*, *SlEDS1*, *SlPAD4*, and *SlPR5*), seven jasmonic acid signaling-related (*SlAOC*, *SlLoxD*, *SlJAZ1*, *SlCOI1*, *SlMYC2*, *SlPI II*, and *SlLapA1*), one ethylene signaling-related (SlERF1) and three non-antioxidant pathway (*SlGST*, *SlAPX*, and *SlP5CS*) genes were selected. **(A)** *SlNPR1*, **(B)** *SlEDS1*, **(C)** *SlPAD4*, **(D)** *SlPR5*, **(E)** *SlAOC*, **(F)** *SlLoxD*, **(G)** *SlJAZ1*, **(H)** *SlCOI1*, **(I)** *SlMYC2*, **(J)** *SlPI II*, **(K)** *SlLapA1*, **(L)** *SlEF1*, **(M)** *SlGST*, **(N)** *SlAPX*, **(O)** *SlP5C5*. Values represent the means ± SD of three independent samples (Student’s *t*-test, *P < 0.05 and **P < 0.01).

To analyze the molecular processes underlying the positive effect of 3-MP on the resistance of tomato fruit to *B. Cinerea*, the transcripts of previously reported stress-related genes involved in jasmonic acid (JA), salicylic acid (SA), ethylene signaling pathway, and non-antioxidant pathway, which are usually chosen as biomarkers for plant stress response, were evaluated in tomato fruit treated with or without 3-MP. As shown in Figure 8, the expression level of *SlNPR1*, *SlPR5*, *SlJAZ1*, *SlCOI1*, *SlPI II*, and *SlLapA1* gene at 24 h in control fruits was higher than that in the fruits treated with 3-MP, while the expression level of remaining genes exhibited no significant difference between control and treatment groups. It should specifically be mentioned that this phenomenon was reversed at 48 h to some extent, as the expression level of *SlNPR1*, *SlJAZ1*, *SlPI II*, *SlGST*, and *SlAPX* gene in control fruits was significantly lower than that in the tomato fruits treated with 3-MP. However, the transcripts of other genes, mainly such as *SlEDS1*, *SlPAD4*, *SlAOC*, *SlLapA1*, and *SlERF1*, were higher in the control fruits when compared to that in the 3-MP-treated fruits. Most importantly, the expression level of most of the genes in the fruits treated with 3-MP for 96 h was significantly higher than that in control fruits, especially *SlEDS1* and *SlPR5* gene, whose transcripts in the 3-MP-treated fruits were 3.85 and 12.6 times higher than that in the control fruits, respectively. These results indicated that various defense pathways were activated in response to *B. cinerea* infection, and the damage caused by *B. cinerea* was greatly alleviated by treatment of 3-MP.

### Effects of 3-Methylthio-1-Propanol on L929, A549, and 4T1 Cell Viability

**FIGURE 9:**
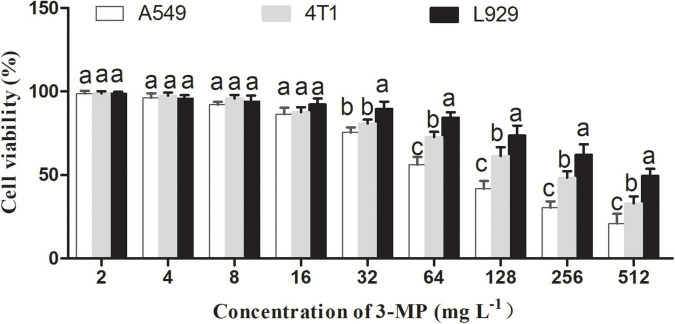
Effects of 3-MP on L929, A549, and 4T1 cell viability. Values are the means ± SD of three biological replicates. The different lowercase letter means statistically significant (Duncan’s multiple range test).

To our knowledge, the existed reports indicated that 3-MP mainly comes from fruit and beer and is mainly used as a food flavoring additive (Moreira et al., 2011). However, there is no report to evaluate whether 3-MP is harmful to humans. As shown in Figure 9, 3-MP exhibited the inhibitory effects on two different tumor cells (i.e., A549 and 4T1) compared to the normal cell (i.e., L929), and this inhibitory effect was more significant with its increasing concentration. According to the result of cell viability, the IC50 value of 3-MP on L929, A549, and 4T1 was 499.19, 91.12, 239.94 μg ml^–1^, respectively. Compared to 4T1 cells, 3-MP showed higher effectiveness in inhibiting A549 proliferation from the perspective of cell level, and this phenomenon deserved further study.

## Discussion

The food-borne sulfur-containing spice compounds are the ideal substitutes for chemical fungicides due to their excellent characteristics of antioxidant, anticancer, and antimicrobial activity (Schöller et al., 2002; Hongman et al., 2018; Xie et al., 2020). However, the existed studies on sulfur-containing spice compounds are not matched their important roles, it is necessary to explore some new sulfur-containing spice compounds to be chosen. 3-MP was found widely in many foods as a natural sulfur-containing spice compound (Etschmann et al., 2008; Moreira et al., 2010) and also used as an important food flavoring additive (Zhou et al., 2021). However, its biological control effect and mechanism on fungal diseases have not been explored yet. In this study, we demonstrated that 3-MP has excellent antifungal activity against *B. cinerea* by using *in vitro* and *in vivo* experiments, and further study showed 3-MP can affect the infection and growth of *B. cinerea*, as well as the defense systems of tomato fruit.

Mycelium is the main form of *B. cinerea* infecting plant hosts (Bika et al., 2021), and the germination of spores is necessary for the formation of the next round of infection (Castillo et al., 2017). The inhibition of mycelial growth and spore germination is an important antifungal mechanism of fungicides or compounds (Pande et al., 2006; Romanazzi et al., 2016). In our current research, 3-MP effectively inhibited the mycelial growth and spore germination of *B. cinerea*. This inhibitory effect of 3-MP was positively correlated with the concentration of 3-MP. This dose dependence is consistent with the previous studies of other compounds such as hinokitiol and epsilon-poly-L-lysine (Dou et al., 2021).

Importantly, spores can contribute not only to a new round of infection but also to the long-distance transmission of pathogens (Etxebeste et al., 2019). It is a very important way to inhibit the spread and infection of the pathogen by destroying the cell membrane structure and reducing the spore viability. Many studies indicated that various antifungal agents can destroy the integrity of the cell membrane, which would lead to the leakages of cytoplasmic contents and finally result in a decrease in vitality and even death of the spores (Guo et al., 2020; Wang et al., 2020). Similarly, our results showed that 3-MP treatment has greatly impaired the spore viability and membrane integrity of *B. cinerea* as well as increased the leakage of cellular components such as the content of nucleic acids, protein, and the MDA content in *B. cinerea.* This damage results in cell dysfunction and the suppression of mycelial growth and spore germination, all of which reduce pathogenicity.

Further studies have shown that 3-MP can differentially downregulate the expression level of key genes related to pathogenicity and growth of the pathogen. *BcPG1*, *BcBMP1*, *BcPLC1*, *BcPLS1*, and *BcPME1* are key genes in determining the infection and growth of *B. cinerea*, and many studies demonstrated that the knockout of these genes can greatly affect the virulence and infection process of *B. cinerea* (Zheng et al., 2000; Valette-Collet et al., 2003; Gourgues et al., 2004; Brito et al., 2006; Schumacher et al., 2008; Wang et al., 2020). Given this, the transcript level of these genes was evaluated, and our data indicated that 3-MP can differentially downregulate the expression level of the five genes, suggesting that the downregulation of these genes might partly contribute to the antifungal effects of 3-MP.

To tolerate the environmental stimulus, a series of alterations occur at molecular or physiological levels in host tissues, mainly such as the activation of ROS regulatory systems and various hormone signaling pathways (Suzuki, 2016). The excessive ROS accumulation can disrupt the redox homeostasis and then lead to oxidative damage to the host cell, and many reports indicated that necrotrophic pathogens would stimulate to generate the masses of ROS into host tissues to induce cell death (Glazebrook, 2005; van Kan, 2006; Sun et al., 2021). POD, SOD, and CAT are the pivotal antioxidant enzymes that demonstrated to play important roles in redundant ROS-scavenging, and many previous studies showed that antifungal agents or biocontrol strains can greatly improve the activity of these enzymes to cope with the invasion of pathogens (Yan et al., 2018; Liu et al., 2019; Sun et al., 2021). Similarly, our results demonstrated that the activity of POD, SOD, and CAT in the fruits of the 3-MP-treated group was still higher than that in the control group at all the monitoring times, which were well reflected by the MDA data, as the MDA content in the fruits treated by 3-MP was significantly lower than that in the control fruit (Figure 7). These results indicate that the relatively high activity of POD, SOD, and CAT in the 3-MP-treated group might partly contribute to tomato fruit that suffers less oxidative damage. SA and JA are important hormones in plant defense responses, and SA and JA signaling pathways would be activated in response to pathogen infections (Zhang et al., 2016; Arnao and Hernández-Ruiz, 2017; Sun et al., 2021). In this study, the expression of most of the stress-related genes was first induced in the control fruit, while their expression in 3-MP fumigation treatment exhibited a significant delay. However, this phenomenon was broken at 96 h subjected to *B. cinerea* infection, as the transcript of most of the genes in the fruits by 3-MP fumigation treatment was significantly higher than that in the control fruits (Figure 8). We speculated that these phenomena might attribute to the faster colonization and higher accumulation of *B. cinerea* in the control fruits than that in the 3-MP-treated group, which would activate the various host defense routes to resist the invasion of *B. cinerea*. Also, with the accumulation of *B. cinerea*, it reached maximized at 96 h in the control fruits, which would lead to the collapse of the defense routes in the control tomato. Overall, these results indicated that both the antioxidant enzyme system and the defense signaling pathways in host tissues were activated in response to *B. cinerea* infection, and the antioxidant enzyme system might partly contribute to alleviate the damage caused by *B. cinerea* in the fruits of the 3-MP-treated group.

In this study, we also evaluated the safety of 3-MP by the cell survival assay. It was considered that the material had no obvious toxicity to cells when the cell survival rate is more than 80% (Atkinson et al., 2019). The results of cytotoxicity demonstrated that 3-MP can specifically inhibit the proliferation of tumor cells and had no toxicity to normal cells in a certain range of concentration (0–89.93 mg L^–1^). Similarly, apoptin specifically killed cancer cells but had no toxicity to normal cells (Cui et al., 2020). This indicated that 3-MP had the potential to become a targeted drug for inhibiting cancer cells. Furthermore, the content of 3-MP in the fumigated tomato fruit (1.6–2.24 mg L^–1^) (Supplementary Figure 1) was far below the threshold of cytotoxicity (89.93 mg L^–1^) (Figure 9), and it was also lower than that in most wines (4.91 mg L^–1^) (Fedrizzi et al., 2007; González-Álvarez et al., 2012). These results indicated that 3-MP may not damage our bodies as a kind of food additive and also indirectly proved that 3-MP was a safe compound that could be used during postharvest storage.

## Conclusion

Our study revealed the excellent antifungal roles of sulfur-containing spice compound 3-MP in both *in vitro* and *in vivo* conditions. The mechanism involved in the antifungal effect of 3-MP against *B. cinerea* may be attributed to its capability to inhibit mycelial growth and spore germination, impair the spore viability and membrane integrity, and downregulate the disease-related genes. In addition, the antioxidant enzyme system and the defense signaling pathways in host tissues might also partly contribute to the antifungal activity of 3-MP. Most importantly, the cytotoxicity assay revealed that 3-MP had no toxicity to normal cells in a certain concentration range. To our knowledge, this is the first report showing the potential of 3-MP as an antifungal agent against gray mold on tomato fruit, and further research on formulations and application methods for developing 3-MP as a new eco-friendly fungicide is necessary.

## Data Availability Statement

The original contributions presented in the study are included in the article/Supplementary Material, further inquiries can be directed to the corresponding authors.

## Author Contributions

SF, WL, and YJ contributed to the experimental design and operation, data analysis, and writing. YC, RM, JD, and QL contributed to the experiment operation and data analysis. TY, LJ, and XY contributed to the suggestion, editing, and adjustment of the format. ZL and WJ contributed to the experimental design, supervision, writing and editing, and funding acquisition. All authors contributed to the article and approved the submitted version.

## Conflict of Interest

The authors declare that the research was conducted in the absence of any commercial or financial relationships that could be construed as a potential conflict of interest.

## Publisher’s Note

All claims expressed in this article are solely those of the authors and do not necessarily represent those of their affiliated organizations, or those of the publisher, the editors and the reviewers. Any product that may be evaluated in this article, or claim that may be made by its manufacturer, is not guaranteed or endorsed by the publisher.

## Footnotes

1
http://www.njjcbio.com/
