# Supplementary material for: Biocontrol Effect and Possible Mechanism of Food-Borne Sulfide 3-Methylthio-1-Propanol Against Botrytis cinerea in Postharvest Tomato
Source: Front Plant Sci. 2021 Dec 14;12:763755. doi: 10.3389/fpls.2021.763755 (PMC8713891; doi:10.3389/fpls.2021.763755)
Supplement: Supplementary file 1 [file Data_Sheet_1.docx]

**Table S1 Primers sequence used in *q*PCR experiments.**

| **Gene Name Primer sequence (5’-3’)** |
| --- |
| *SlNPR1* F: CCCTGGCTAGCATGAGGAAG  R: AGAGAGAGCCCTAAGCCGAT  *SlEDS1* F: GGAATTGAAGTCAGAGATGAGCTAA  R: AAAGTTCCAGCAAAAGCAAAAA  *SlPAD4* F: CCGTGATCAGATGGTAGAAATAATG  R: CGGCAGAGAAGCCAGAGAGT  *SlPR5* F: TCCTTGCCTTTGTGACTT  R: ACCTCTTCCAGCACCATC  *SlAOC* F: CCTCTGCTGCTCTTAGAACCATT  R: CTGGAAGCAGTAGTAGAGGTGGTAG  *SlLoxD* F: GACTGGTCCAAGTTCACGATCC  R: ATGTGCTGCCAATATAAATGGTTCC  *SlJAZ1* F: CGAGACGGAATTCACTTACAAGA  R: TGAGCACCTAATCCCAACCAT  *SlCOI1* F: GGATGCTTCTGGGATACGTT  R: TGGATGCTCCGAGACTACAG  *SlMYC2* F: GGAGGCGAAGACTCTGAACATT  R: GCTGGCTTTCTACCTCGCTTC  *SlPI II* F: TGCCTATTCAAGATGTCCCCGTT  R: GCCTTGGGTTCATCACTCTCTCC  *SlLapA1* F: CATAGCAACTGGAGTTGTGC  R: CCTGCACATACATGTTCTGC  *SlERF1* F: ATTGGAGTTAGAAAGAGGCCAT  R: CTCATTGATAATGCGGCTTG  *SlGST* F: GCAAGCCCATTTGTGAGTCT  R: TGCTGACCCCTTATCATCG  *SlAPX* F: ACGATGATATTGTGACACTCTTCCA  R: AAGCGATGAAACCACAAAAACA  *SlP5CS* F: TGCTGTAGGTGTTGGTCGTCA  R: TGCCATCAAGCTCAGTTTGTG  *BcPG1* F: GCCAATATGGTTCAACTTCTC  R: GAACCAACATCGAAAGCATC  *BcPLS1* F: CCCGTTCCATTCTTCTCATC  R: GCTCCTACTATTGCCAGTGT  *BcPME1* F: TGGGACGTCCATGGAGAAAC  R: TGATGCCGGAACTGAAGGTT  *BcPLC1* F: TTACCACTGGCTACATACACTG  R: CCGTTTGTGTCGTAAAGAGG  *BcBMP1* F: GGCTATTGATGTCTGGTCTGTTGG  R: CAAGAATGAGTGTGAGTTGGTGGT  *BcActin* F: CTCTATTCAAGCCGTCCTCTCC  R: TAATCAGTCAAATCACGACCAGC  *SlUBI* F: GCCGACTACAACATCCAGAAGG  R: TGCAACACAGCGAGCTTAACC |


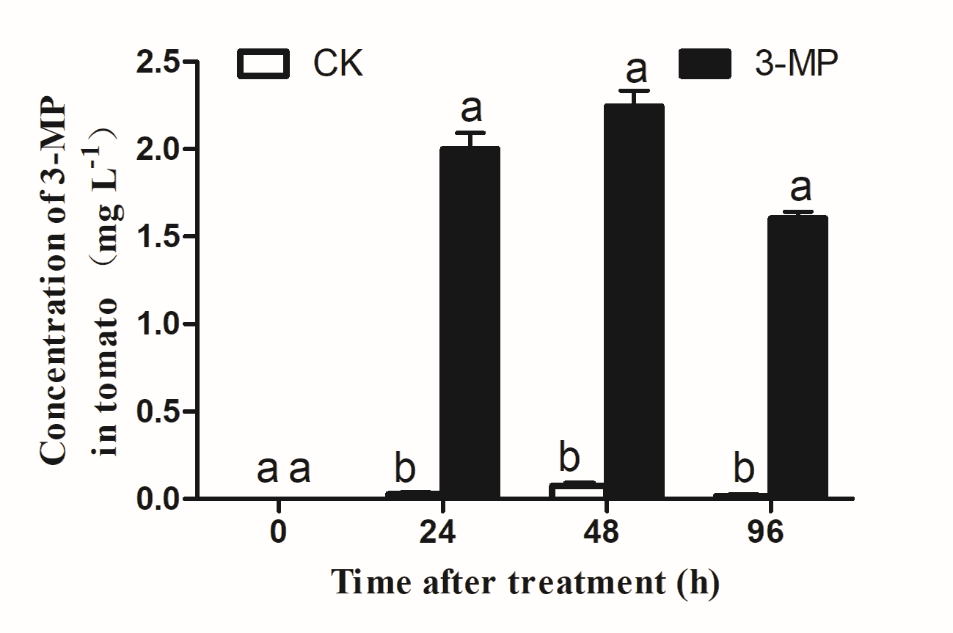


**Fig. S1.** Determination of 3-MP content in fumigated tomato fruit. Values are the means ± SD of three biological replicates. The different lowercase letter means statistically significant (Student’s t-test).

**Supplementary methods**

**Determination of 3-MP.** The content of 3-MP in tomato tissue was measured based on the method of previous studies ([Xu et al., 2018](#_ENREF_1)) with some improvement. 3 × 10^-4^ kg powdered sample and 200 μL of MTBE with 0.25 ng μL^-1^ tetradecane as an internal standard were added into a tube. The tube was stored at room temperature for 2 h, and the MTBE phase was then collected for the subsequent analysis by GC-MS. 1 μL samples were separated and analyzed using GC-MS system (GC, Agilent Technologies 7890B GC System; MS, Agilent 5977B GC/MSD) equipped with the Rxi-5Sil column (30 m × 0.25 mm × 0.25 μm film thickness; Restek). The carrier gas (Helium) was at a flow rate of 1.2 × 10^-3^ L per minute. The injector was used in splitless mode with the inlet temperature set as 250 °C. The column oven was set as 50 °C holdings for 2 min, and then increased 310 °C at a rate of 10 °C per min and finally held for 3 min. Compounds were identified by comparison of mass spectra and retention times with those of the authentic standards, when available, or with known retention indices and mass fragmentation from the literature and NIST library. The measurement of 3-MP was performed based on the internal standard method.

**Reference**

Xu, H.Y., Lybrand, D., Bennewitz, S., Tissier, A., Last, R.L., Pichersky, E., 2018. Production of trans-chrysanthemic acid, the monoterpene acid moiety of natural pyrethrin insecticides, in tomato fruit. Metab Eng 47, 271-278. https://doi.org/10.1016/j.ymben.2018.04.004
